# Occupational disparities in chronic disease prevalence and metabolic covariation: a cross-sectional study of six occupational groups in Lanzhou

**DOI:** 10.3389/fpubh.2026.1950073

**Published:** 2026-09-21

**Authors:** Xiuyun Ma, Liyuan Chen, Lin Wang, Longyu Wang, Linping Wu, Yuanming Wang, Xuexia Ma, Shaochen Su

**Affiliations:** 1 School/Hospital of Stomatology Lanzhou University, Lanzhou, Gansu, China; 2 Lanzhou University First Hospital, Lanzhou, Gansu, China; 3 Lanzhou University Second Hospital, Lanzhou, Gansu, China

**Keywords:** age-standardized prevalence, chronic diseases, logistic regression, metabolic comorbidity network, occupational population

## Abstract

**Objectives:**

To investigate the differences in chronic disease prevalence and underlying metabolic profiles among working-age individuals across six typical occupational groups, identify occupation-related associated factors, and provide epidemiological evidence for occupation-specific precise prevention and control of chronic diseases.

**Methods:**

A cross-sectional study design was adopted. A total of 1,473 on-the-job physical examinees aged 18–64 who received physical examination at the Physical Examination Center of the First Hospital of Lanzhou University from August 2024 to August 2025 were retrospectively included. Structured questionnaires were distributed via targeted online channels to collect sociodemographic characteristics and behavioral and lifestyle data. Physical examinations and laboratory tests were performed to obtain 16 health indicators. Age-standardized prevalence (ASR) of five chronic diseases (hypertension, diabetes mellitus, hyperlipidemia, hyperuricemia, and obesity) was calculated using the direct standardization method, with the age composition of the full study sample as the standard population. Multivariate logistic regression analysis was conducted to assess the independent associations between various factors and chronic diseases, and Spearman rank correlation analysis was used to evaluate the strength of associations between health indicators.

**Results:**

Occupational disparities in the prevalence of hyperlipidemia and hyperuricemia were the most prominent. After age standardization, the prevalence of hyperlipidemia in police officers (ASR = 0.435, 95% CI 0.368–0.502) and software engineers (ASR = 0.418, 95% CI 0.366–0.470), as well as the prevalence of hyperuricemia in firefighters (ASR = 0.381, 95% CI 0.315–0.447) and police officers (ASR = 0.332, 95% CI 0.267–0.396) were all significantly higher than those in healthcare workers (ASR = 0.218 for hyperlipidemia and 0.143 for hyperuricemia, respectively), with no overlap in 95% CIs across occupational groups. Multivariate logistic regression analysis showed that police officers (OR = 2.499, 95% CI 1.409–4.433) and software engineers (OR = 2.531, 95% CI 1.007–6.361) were independent associated factors for hyperlipidemia; police officers (OR = 3.425, 95% CI 1.821–6.441), firefighters (OR = 4.143, 95% CI 1.916–8.955), and software engineers (OR = 2.957, 95% CI 1.136–7.696) were independent associated factors for hyperuricemia. No statistically significant occupational disparities were observed for hypertension, diabetes mellitus, and obesity. Correlation analysis showed that triglyceride, high-density lipoprotein cholesterol, body mass index, uric acid and alanine aminotransferase formed a moderate correlation cluster (|*r*| = 0.44–0.54), body mass index was widely correlated with a variety of metabolic indicators (*r* = 0.39–0.51), and uric acid was strongly correlated with creatinine (*r* = 0.64).

**Conclusion:**

Police officers, firefighters, and software engineers are occupational groups with elevated prevalence of hyperlipidemia and hyperuricemia. Age standardization is a necessary methodological step for cross-occupational health comparisons. Obesity occupies a core hub position in the metabolic comorbidity network. These findings provide reference evidence for developing occupation-specific, tiered precise prevention and control strategies for chronic diseases.

## 1 Background

Cardiovascular diseases, diabetes, obesity, and related metabolic disorders have become core contributors to the global burden of disease (1–3). Data from the Global Burden of Disease Study 2021 (GBD 2021) and associated analytical reports show that disability-adjusted life years (DALYs) attributable to cardiovascular diseases and other chronic non-communicable diseases driven by metabolic associated factors exhibited an overall increasing trend globally during 1990–2021 (4). Notably, the growth of this burden was particularly pronounced in low and middle-income countries (LMICs), and exhibited a syndemic pattern characterized by propagation predominantly among younger adults (particularly those under 40 years of age) (5, 6). As the core driver of social productivity, the burden of chronic diseases in the working-age population not only reduces individual healthy life expectancy but also imposes substantial economic and social costs; thus, identifying modifiable associated factors in this demographic holds critical public health significance.

Occupations not only serve as a source of income and social security, but also constitute a key component of the individual lifelong exposome (the aggregate of all environmental exposures across an individual’s lifetime), exerting profound impacts on metabolic and cardiovascular health via multiple pathways spanning physiological, psychological, and environmental domains (7, 8). Prior studies have mostly focused on internal exposure-response relationships within specific occupations, while cross-occupational comparisons anchored in a unified analytical framework remain scarce for occupations with markedly divergent social functions, work rhythms, and exposure profiles (9, 10). Notably, in the Chinese context, systematic comparative analyses of disparities in metabolic disease burden and their underlying drivers across typical occupational groups (including healthcare workers, teachers, police officers, firefighters, software engineers, and financial practitioners) remain limited. Given the increasingly prominent syndemic trend of metabolic diseases among young adults globally, and the substantial contribution of metabolic associated factors to the burden of cardiovascular disease, addressing this research gap is essential for developing precision-oriented occupational health intervention strategies (5, 11).

Building on this, the present study targeted working-age populations from six typical occupational groups: healthcare workers, teachers, police officers, firefighters, software engineers, and financial practitioners. Data on basic sociodemographic characteristics, lifestyle factors, and 16 clinical examination metrics were collected via a cross-sectional survey. We systematically characterized differences in sociodemographic and health profiles among occupational groups receiving routine health checkups in Lanzhou, calculated age-standardized prevalence rates of five categories of metabolism-related chronic diseases to quantify inter-occupational disease burden, and applied multivariable logistic regression and Spearman correlation networks to separately investigate the independent associations of occupational, behavioral, and social factors with chronic disease prevalence, as well as the covariation patterns of metabolic indicators, to provide evidence for precision prevention and control strategies stratified by occupation.

## 2 Study population and methods

### 2.1 Study design and study population

This study is a single-center retrospective cross-sectional study targeting a health check-up population, with study participants being specific occupational groups who received routine health examinations at the Physical Examination Center of the First Hospital of Lanzhou University from August 2024 to August 2025. The study aims to investigate disparities in chronic disease prevalence and patterns of health indicators across different occupational groups within this specific population. The Physical Examination Center of the First Hospital of Lanzhou University was designated as the sampling site. Leveraging its advantage of maintaining health records covering the working population of Lanzhou, we screened resident target occupational groups in the city to enable efficient sampling. This study employed a convenience sample-based random sampling approach. In the first stage, all records corresponding to the six predefined target occupational groups (healthcare workers, teachers, police officers, firefighters, software engineers, and financial practitioners) were screened from the overall health examination database of the Physical Examination Center of the First Hospital of Lanzhou University, based on occupational information recorded on health examination registration forms, to generate a preliminary occupational grouping roster. In the second stage, simple random sampling was performed for each occupational grouping list using computer-generated random numbers. Finally, questionnaire links were distributed in a targeted manner via telephone notifications, physical examination WeChat groups, and other social media channels. With ethical approval (LZUKQ-2026-052, granted in March 2026), this study retrospectively extracted health examination records of the occupational population from the Health Examination Center of The First Hospital of Lanzhou University, with records collected between August 2024 and August 2025. Online questionnaires were subsequently distributed and collected from this occupational population starting in April 2026.

To ensure data quality, strict screening criteria were established for this study. A total of 1,473 eligible participants were ultimately included in the analysis, with each of the 6 occupational groups comprising 150–400 participants. The specific inclusion criteria were as follows: 1. Aged within the working-age range of 18–64 years; 2. Have been continuously employed in Gansu Province for ≥6 months; 3. Have complete physical examination records, signed informed consent forms, and completed the questionnaire; 4. Belong to one of the six predefined occupational categories: healthcare workers, teachers, police officers, firefighters, software engineers, and financial workers. The exclusion criteria were as follows: 1. Aged outside the working-age range of 18–64 years; 2. Individuals with severe missing information in their physical examination data; 3. Questionnaires with severe incompleteness, logical contradictions, or other quality issues. This study protocol was approved by the Ethics Review Committee of the School and Hospital of Stomatology, Lanzhou University, with the approval number lzukq-2026-052. The approval covers the retrospective collection of data from the hospital’s physical examination center starting from March 2026, as well as questionnaire distribution and data analysis. As this study only involved anonymized data analysis, the ethics committee waived the requirement for participants to sign informed consent forms.

Sample size was determined using Hsieh et al. (12) logistic regression method. Hyperlipidemia was chosen as the primary endpoint because it exhibited the largest occupational disparities (ASR 21.8–43.5%) and it’s smallest significant OR (≈2.5) ensured conservative power for all outcomes. Anticipated prevalence (35%) was based on national (NHCC, 2024: 35.6%). With *α* = 0.05, power = 0.80, OR = 2.5, and *ρ*^2^ = 0.2, the required total sample was ~1,200 (200/group). Our achieved sample (*n* = 1,473) exceeds this. Post-hoc power >80% for all significant ORs (2.5–4.1). Sensitivity analyses excluding the two smallest groups (teachers *n* = 156, financial workers *n* = 193) did not alter results (Supplementary Table S1, Figure 1).

**Figure 1:**
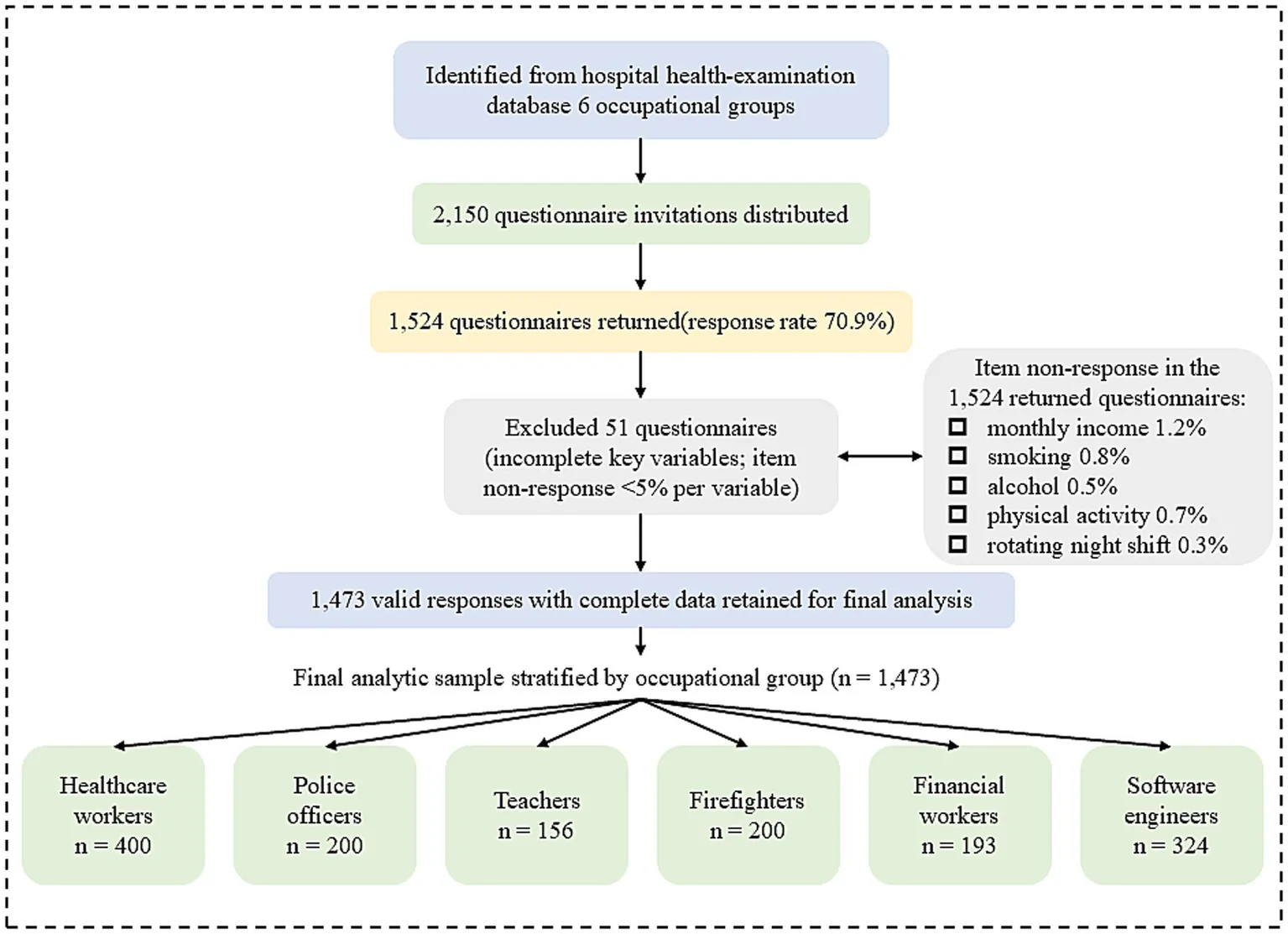
STROBE flow diagram.

### 2.2 Data collection

#### 2.2.1 Basic characteristics survey

Sociodemographic characteristics and behavioral and lifestyle factors were collected via a self-developed structured questionnaire, including gender, age (stratified into three age groups: 18–34 years, 35–49 years, and 50–64 years), occupation (healthcare workers, teachers, police officers, firefighters, software engineers, and financial works), educational attainment (high school/secondary vocational school or below, college/associate degree, and graduate degree or above), monthly average income (<5,000 RMB, 5,000–10,000 RMB, 10,000–15,000 RMB, and ≥15,000 RMB), smoking status (never smokers, former smokers, and current smokers), drinking status [non-regular drinking/regular drinking, with regular drinking defined as consuming alcohol ≥50 g per occasion at least twice per week over the past year(Chinese Dietary Guidelines 2022)], physical activity frequency (weekly physical activity duration ≥150 min vs. <150 min), and rotating night shift work (yes/no, defined as working through the entire night until the following morning, and having completed at least one night shift per month over the past 6 months). Data collection adhered to the principle of anonymization to protect participant privacy.

#### 2.2.2 Physical examination and laboratory testing

Height and weight were measured using standardized methods, and body mass index (BMI) was calculated as weight (kg) divided by the square of height (m) [formula: BMI = weight (kg)/height (m)^2^]. Blood pressure was measured using an Omron electronic sphygmomanometer, with three consecutive measurements taken from the right upper arm in a resting sitting position, and the mean value was recorded. Fasting venous blood samples were collected after ≥8 h of fasting to measure the following 16 indicators: total cholesterol (TC), triglycerides (TG), high-density lipoprotein cholesterol (HDL-C), low-density lipoprotein cholesterol (LDL-C), uric acid (UA), creatinine (Cr), blood urea nitrogen (BUN), alanine aminotransferase (ALT), aspartate aminotransferase (AST), total bilirubin (TBIL), direct bilirubin (DBIL), indirect bilirubin (IBIL), fasting plasma glucose (FPG), systolic blood pressure (SBP), diastolic blood pressure (DBP), and BMI. All tests were performed by the clinical laboratory department of the Physical Examination Center of the First Hospital of Lanzhou University.

#### 2.2.3 Diagnostic criteria for chronic diseases

Hypertension: Defined as systolic blood pressure (SBP) ≥ 140 mmHg and/or diastolic blood pressure (DBP) ≥ 90 mmHg, or a prior physician-confirmed diagnosis with ongoing antihypertensive treatment. Diabetes: Defined as fasting plasma glucose (FPG) ≥ 7.0 mmol/L, or OGTT 2 h plasma glucose ≥11.1 mmol/L, or HbA1c ≥ 6.5%, or a prior physician-confirmed diagnosis with ongoing hypoglycemic medication use. Dyslipidemia: Defined as total cholesterol (TC) ≥ 6.2 mmol/L, or triglycerides (TG) ≥ 2.3 mmol/L, or low-density lipoprotein cholesterol (LDL-C) ≥ 4.1 mmol/L, or high-density lipoprotein cholesterol (HDL-C) < 1.0 mmol/L, or a prior physician-confirmed diagnosis of dyslipidemia. Hyperuricemia: Defined as uric acid (UA)>420 μmol/L in males and postmenopausal females, or>360 μmol/L in premenopausal females. Obesity: Defined as body mass index (BMI) ≥ 28 kg/m^2^.

### 2.3 Statistical methods

#### 2.3.1 Descriptive statistics

Descriptive analyses were conducted to examine sociodemographic and health indicators across all occupational groups. Continuous data are presented as mean ± standard deviation (SD), while categorical data are reported as frequency (percentage) *n* (%),and between-group comparisons were performed using the Pearson *χ*^2^ test or Fisher’s exact test, with the latter applied when more than 20% of cells had expected frequencies <5. All statistical tests were two-sided, and a significance threshold of *α* = 0.05 was used for all analyses.

Of 2,150 questionnaire invitations, 1,524 were returned (response rate 70.9%). After excluding 51 questionnaires with incomplete key variables (item non-response rate <5% for any single variable), 1,473 valid responses with complete data on all variables were retained for the final analysis. Item non-response rates in the initial 1,524 returned questionnaires were: monthly income 1.2%, smoking status 0.8%, alcohol consumption 0.5%, physical activity 0.7%, and rotating night shift 0.3%. All 16 health indicators from electronic health records had 0% missingness. Little’s MCAR test indicated that missingness was completely at random (*χ*^2^ = 12.34, df = 10, *p* = 0.26). The final analytic sample contained no missing data, complete-case analysis was used for all logistic regression models.

#### 2.3.2 Age-standardized prevalence

Age-standardized prevalence of chronic diseases for each occupational group was calculated using the direct standardization method. The standard population was derived from the age distribution of the full study sample, with participants stratified into three age brackets: 18–34 years (54.9%), 35–49 years (32.8%), and 50–64 years (12.3%). Crude prevalence reflects the observed disease burden in each occupational group, whereas age-standardized rates eliminate the confounding effect of differences in age structure, enabling a more accurate assessment of the association between occupational factors and chronic disease burden. The calculation formula is as follows:

$${\mathrm{ASR}}_{\mathrm{jk}}=\sum_{i=1}^{3}(w_{i}\times p_{\mathrm{ijk}})$$

Where *w*_i_ denotes the population weight of the *i*^th^ age group in the standard population, and $p_{\mathrm{ijk}}=\frac{a_{\mathrm{ijk}}}{n_{\mathrm{ijk}}}$ represents the crude prevalence of the *i*^th^ age group for the given occupation.

The 95% confidence interval (95% CI) of the age-standardized rate was calculated using the Greenspoon–Levy variance approximation method; the variance calculation formula is as follows:

$$\mathrm{Var}({\mathrm{ASR}}_{\mathrm{jk}})=\sum_{i=1}^{3}w_{i}^{2}\times \frac{p_{\mathrm{ijk}}(1-p_{\mathrm{ijk}})}{n_{\mathrm{ijk}}-1}$$

95%CI: ${\mathrm{ASR}}_{\mathrm{jk}}\pm 1.96\times \sqrt{\mathrm{Var}({\mathrm{ASR}}_{\mathrm{jk}})}$ , corresponding to a lower limit of ${\mathrm{ASR}}_{\mathrm{jk}}-1.96\times \sqrt{\mathrm{Var}({\mathrm{ASR}}_{\mathrm{jk}})}$ and upper limit of ASR+1.96×SE.

To assess the robustness of our findings to the choice of standard population, age-standardized prevalences were recalculated using the 18–64 working-age population structure of Gansu Province from the 2020 Chinese Population Census (7th National Census) as an external standard. The three age groups (18–34, 35–49, 50–64 years) accounted for 34.13, 32.20, and 33.68% of the provincial 18–64 working-age population, respectively. The recalculated ASRs showed the same occupational ranking as the internal-standard ASRs for all five chronic diseases. Full results are reported in Supplementary Table S3.

#### 2.3.3 Multivariate logistic regression analysis

The status of each chronic disease (hypertension, diabetes, hyperlipidemia, hyperuricemia, and obesity) was set as the dependent variable (0 = no disease, 1 = prevalent disease), and five separate binary logistic regression models were constructed, respectively. The independent variables included sex, age group (dummy variable), occupation (dummy variable), educational attainment (dummy variable), monthly income (dummy variable), smoking status (dummy variable), alcohol consumption, physical activity, and rotating night shift work. Among these variables, the categorical variables (occupation, age group, educational attainment, monthly income, and smoking status) were coded as dummy variables using indicator contrast, with reference groups set as healthcare workers, 18–34 years, high school or technical secondary school and below, <5,000 RMB per month, and never smokers, respectively. Binary variables (sex, alcohol consumption, physical activity, and rotating night shift) were directly included in the models, with the category assigned a lower code value serving as the reference.

Variable selection was performed using the Enter method, which included all candidate variables in the model to avoid potential variable omission and biased parameter estimation caused by stepwise regression. Model goodness of fit was assessed using the Hosmer–Lemeshow test (*p* > 0.05 indicates good fit), and model explanatory power was evaluated using the Nagelkerke R^2^. Results are presented as odds ratios (OR) with corresponding 95% confidence intervals (CI). The significance level was set at α = 0.05 (two-sided). Additionally, marginally significant results (*p* < 0.10) are reported as indicative of trends.

Multicollinearity was assessed using Variance Inflation Factors (VIF) derived from a linear regression model containing the same independent variables as the logistic regression models. VIF values < 5 were considered indicative of no serious multicollinearity (Supplementary Table S2).

#### 2.3.4 Spearman correlation analysis

As the 16 physical examination indicators mostly follow skewed distributions (confirmed by the Shapiro–Wilk test), Spearman rank correlation coefficients were used to assess the strength of pairwise associations between indicators. The interpretation criteria of correlation coefficient |r_s_| were as follows: |r_s_| < 0.3 as weak correlation, 0.3 ≤ |r_s_| < 0.5 as moderate correlation, and |r_s_| ≥ 0.5 as strong correlation. Correlation heatmaps were generated using R packages.

#### 2.3.5 Statistical software

All statistical analyses and plotting were performed using SPSS 27.0 (IBM Corp., Armonk, NY, United States), GraphPad Prism 10, R packages, and Microsoft Excel.

## 3 Results

### 3.1 Basic characteristics of study participants

A total of 1,473 participants were enrolled in this study (Table 1). Of these individuals, males accounted for 63.07%, the 18–34 years age group accounted for 54.85%, those with a university/college degree accounted for 55.13%, participants with a monthly income of 10,000–15,000 RMB accounted for 64.02%, never smokers accounted for 63.27%, non-active drinkers accounted for 78.34%, those with weekly physical activity duration exceeding 150 min accounted for 53.22%, and individuals whose work requires rotating/night shifts accounted for 52.7%. The study cohort comprised 156 teachers, 200 police officers, 193 financial workers, 200 firefighters, 324 software engineers, and 400 healthcare workers. Sociodemographic characteristics and occupational distribution of these six occupational groups are summarized in Table 1.

**Table 1:** Basic characteristics of participants across different occupational groups [*n*(%)].

Variables	Healthcare workers (*n* = 400)	Teachers (*n* = 156)	Police officers (*n* = 200)	Firefighters (*n* = 200)	Financial workers (*n* = 193)	Software engineers (*n* = 324)	Total (*n* = 1,473)	*χ* ^2^	*P*
Gender								314.796	<0.001
Male	141 (35.3)	81 (51.9)	166 (83.0)	199 (99.5)	102 (52.8)	240 (74.1)	929 (63.1)		
Female	259 (64.8)	75 (48.1)	34 (17.0)	1 (0.5)	91 (47.2)	84 (25.9)	544 (36.9)		
Age/Year								326.569	<0.001
[18,34]	223 (55.8)	33 (21.2)	77 (38.5)	164 (82.0)	79 (40.9)	232 (71.6)	808 (54.9)		
[35,49]	151 (37.8)	76 (48.7)	71 (35.5)	35 (17.5)	62 (32.1)	88 (27.2)	483 (32.8)		
[50,64]	26 (6.5)	47 (30.1)	52 (26.0)	1 (0.5)	52 (26.9)	4 (1.2)	182 (12.3)		
Education level								400.453	<0.001
High school/vocational school or below	30 (7.5)	12 (7.7)	38 (19.0)	63 (31.5)	6 (3.1)	5 (1.5)	154 (10.5)		
Tertiary education qualification	113 (28.2)	104 (66.7)	137 (68.5)	128 (64.0)	113 (58.5)	217 (67.0)	812 (55.1)		
Master’s degree or above	257 (64.3)	40 (25.6)	25 (12.5)	9 (4.5)	74 (38.3)	102 (31.5)	507 (34.4)		
Monthly income/RMB								146.935	<0.001
<5,000	52 (13.0)	23 (14.7)	19 (9.5)	12 (6.0)	11 (5.7)	26 (8.0)	143 (9.7)		
[5000–10,000)	181 (45.3)	98 (62.8)	144 (72.0)	163 (81.5)	155 (80.3)	202 (62.3)	943 (64.0)		
[10000–15,000)	129 (32.3)	27 (17.3)	21 (10.5)	23 (11.5)	14 (7.3)	85 (26.2)	299 (20.3)		
≥15,000	38 (9.5)	8 (5.1)	16 (8.0)	2 (1.0)	13 (6.7)	11 (3.4)	88 (6.0)		
Smoking status								25.270	0.005
Never smokers	282 (70.5)	92 (59.0)	113 (56.5)	114 (57.0)	120 (62.2)	211 (65.1)	932 (63.3)		
Former smokers	21 (5.3)	16 (10.3)	14 (7.0)	25 (12.5)	19 (9.8)	24 (7.4)	119 (8.1)		
Current smokers	97 (24.3)	48 (30.8)	73 (36.5)	61 (30.5)	54 (28.0)	89 (27.5)	422 (28.6)		
Alcohol consumption								11.135	0.049
Non-regular drinkers	321 (80.3)	121 (77.6)	148 (74.0)	167 (83.5)	139 (72.0)	258 (79.6)	1,154 (78.3)		
Regular drinkers	79 (19.8)	35 (22.4)	52 (26.0)	33 (16.5)	54 (28.0)	66 (20.4)	319 (21.7)		
Physical activity								256.946	<0.001
≥150 min/w	177 (44.3)	54 (34.6)	163 (81.5)	184 (92.0)	75 (38.9)	131 (40.4)	784 (53.2)		
<150 min/w	223 (55.8)	102 (65.4)	37 (18.5)	16 (8.0)	118 (61.1)	193 (59.6)	689 (46.8)		
Rotating night shift work								1322.672	<0.001
Yes	384 (96)	0 (0)	177 (88.5)	181 (90.5)	13 (6.7)	21 (6.5)	776 (52.7)		
No	16 (4)	156 (100)	23 (11.5)	19 (9.5)	180 (93.3)	303 (93.5)	697 (47.3)		

### 3.2 Crude and age-standardized prevalence of chronic diseases

After age standardization (Table 2), the prevalence of chronic diseases exhibited distinct distribution patterns across occupational groups. For hyperlipidemia, the age-standardized prevalence among police officers (0.435, 95% CI 0.368–0.502) and software engineers (0.418, 95% CI 0.366–0.470) was significantly higher than that of healthcare workers (0.218, 95% CI 0.178–0.258), with no overlapping 95% CIs between groups. For hyperuricemia, the age-standardized prevalence among firefighters (0.381, 95% CI 0.315–0.447) and police officers (0.332, 95% CI 0.267–0.396) was also significantly higher than that of healthcare workers (0.143, 95% CI 0.109–0.178). For hypertension, financial workers had the highest age-standardized prevalence (0.179, 95% CI 0.125–0.233), but 95% CIs showed partial overlap across all occupational groups. No statistically significant differences in age-standardized prevalence of diabetes mellitus and obesity were observed across occupational groups. Of note, the crude and age-standardized prevalence of chronic diseases among teachers and firefighters exhibited divergent trends: the crude prevalence among teachers was artificially inflated due to their older age structure, while the crude prevalence among firefighters was underestimated due to their younger age composition, indicating that age structure is a key confounding factor in cross-occupational disease comparisons.

**Table 2:** Age-standardized prevalence of chronic diseases across different occupational groups.

Diseases	Occupations	*n*	CR	ASR	95% CI
Hypertension	Healthcare workers	400	9.8%	8.8%	6.0–11.6%
	Teachers	156	18.5%	16.6%	10.7–22.4%
	Police officers	200	12.0%	10.8%	6.5–15.1%
	Firefighters	200	5.1%	4.6%	1.7–7.5%
	Software engineers	324	10.5%	9.4%	6.2–12.6%
	Finance works	193	20.0%	17.9%	12.5–23.3%
Diabetes	Healthcare workers	400	2.5%	2.2%	0.8–3.7%
	Teachers	156	7.0%	6.3%	2.4–10.1%
	Police officers	200	3.0%	2.7%	0.4–5.0%
	Software engineers	324	2.5%	2.2%	0.6–3.9%
	Finance works	193	4.5%	4.0%	1.2–6.8%
Hyperlipidemia	Healthcare workers	400	24.3%	21.8%	17.8–25.8%
	Teachers	156	41.5%	37.2%	29.8–44.7%
	Police officers	200	48.5%	43.5%	36.8–50.2%
	Firefighters	200	35.0%	31.4%	25.0–37.7%
	Software engineers	324	46.6%	41.8%	36.6–47.0%
	Finance works	193	37.5%	33.6%	27.1–40.2%
Hyperuricemia	Healthcare workers	400	16.0%	14.3%	10.9–17.8%
	Teachers	156	29.5%	26.5%	19.6–33.3%
	Police officers	200	37.0%	33.2%	26.7–39.6%
	Firefighters	200	42.5%	38.1%	31.5–44.7%
	Software engineers	324	34.9%	31.3%	26.3–36.3%
	Finance works	193	30.5%	27.4%	21.1–33.6%
Obesity	Healthcare workers	400	9.3%	8.3%	5.6–11.1%
	Teachers	156	13.0%	11.7%	6.6–16.7%
	Police officers	200	13.6%	12.2%	7.6–16.7%
	Firefighters	200	10.5%	9.4%	5.4–13.5%
	Software engineers	324	12.3%	11.0%	7.6–14.4%
	Finance works	193	13.5%	12.1%	7.5–16.7%

### 3.3 Multivariable logistic regression analysis

Results of multivariable logistic regression analysis are presented in Table 3 and Figure 2. For hyperlipidemia, independent associated factors included the age group of 35–49 years (OR = 1.676, 95% CI 1.099–2.556, *p* < 0.05), police officers (OR = 2.499, 95% CI 1.409–4.433, *p* < 0.01), and software engineers (OR = 2.531, 95% CI 1.007–6.361, *p* < 0.05). For hyperuricemia, independent associated factors included the age group of 50–64 years (OR = 2.081, 95% CI 1.033–4.193, *p* < 0.05), police officers (OR = 3.425, 95% CI 1.821–6.441, *p* < 0.001), firefighters (OR = 4.143, 95% CI 1.916–8.955, *p* < 0.001), and software engineers (OR = 2.957, 95% CI 1.136–7.696, *p* < 0.05). For hypertension, only firefighters (OR = 0.379, 95% CI 0.121–1.193, *p* < 0.10) and active alcohol consumption (OR = 2.285, 95% CI 0.929–5.622, *p* < 0.10) showed borderline statistical significance. No factors reached statistical significance in either the diabetes mellitus or obesity models. The absence of observed diabetes cases at the # footnote marker was attributable to two primary factors: the small sample size in this stratum, and the strong healthy worker effect inherent to the firefighter occupational cohort. The Hosmer-Lemeshow test yielded *p* > 0.05 for all models, and the Nagelkerke R^2^ value ranged from 0.052 to 0.150 across all included models.

**Table 3:** Results of multivariable logistic regression analysis.

Variables (reference)	Hypertension	Diabetes	Dyslipidemia	Hyperuricemia	Obesity
Gender (Female)					
Male	0.807 (0.447 ~ 1.455)	2.143 (0.510 ~ 9.010)	1.224 (0.828 ~ 1.811)	0.792 (0.523 ~ 1.201)	1.012 (0.541 ~ 1.891)
Age (18–34)					
35–49	1.404 (0.748 ~ 2.635)	1.636 (0.376 ~ 7.117)	1.676 (1.099 ~ 2.556)**	1.392 (0.892 ~ 2.173)	0.946 (0.493 ~ 1.818)
50–64	1.765 (0.640 ~ 4.866)	0.893 (0.102 ~ 7.795)	1.720 (0.890 ~ 3.323)	2.081 (1.033 ~ 4.193)**	0.692 (0.257 ~ 1.862)
Occupations (Healthcare workers)					
Teachers	0.934 (0.239 ~ 3.643)	3.272 (0.219 ~ 48.770)	1.582 (0.592 ~ 4.227)	1.582 (0.565 ~ 4.428)	2.420 (0.633 ~ 9.260)
Police officers	0.926 (0.390 ~ 2.198)	1.413 (0.200 ~ 10.001)	2.499 (1.409 ~ 4.433)***	3.425 (1.821 ~ 6.441)****	1.760 (0.742 ~ 4.173)
Firefighters	0.379 (0.121 ~ 1.193)*	0.00 (0.00 ~ 0.00)#	1.321 (0.647 ~ 2.697)	4.143 (1.916 ~ 8.955)****	1.700 (0.570 ~ 5.069)
Financial workers	0.960 (0.257 ~ 3.578)	2.223 (0.158 ~ 31.291)	1.507 (0.581 ~ 3.914)	1.675 (0.622 ~ 4.513)	2.556 (0.703 ~ 9.297)
Software engineers	0.704 (0.190 ~ 2.605)	0.751 (0.055 ~ 10.183)	2.531 (1.007 ~ 6.361)**	2.957 (1.136 ~ 7.696)**	2.043 (0.585 ~ 7.138)
Education level (High school/vocational school or below)					
Tertiary education qualification	0.671 (0.270 ~ 1.667)	2.893 (0.262 ~ 31.983)	0.922 (0.521 ~ 1.630)	1.056 (0.575 ~ 1.942)	1.421 (0.569 ~ 3.552)
Master’s degree or above	0.595 (0.188 ~ 1.880)	3.900 (0.214 ~ 71.206)	0.768 (0.358 ~ 1.645)	1.268 (0.566 ~ 2.844)	1.078 (0.322 ~ 3.602)
Monthly income/RMB (<5,000)					
5,000–10,000	1.484 (0.636 ~ 3.462)	0.282 (0.049 ~ 1.609)	1.175 (0.695 ~ 1.987)	1.422 (0.807 ~ 2.506)	1.079 (0.467 ~ 2.491)
10,000–15,000	0.630 (0.208 ~ 1.909)	0.558 (0.058 ~ 5.379)	1.182 (0.570 ~ 2.453)	0.933 (0.429 ~ 2.030)	1.505 (0.493 ~ 4.592)
>15,000	1.160 (0.355 ~ 3.787)	0.660 (0.061 ~ 7.160)	1.536 (0.646 ~ 3.653)	1.302 (0.523 ~ 3.244)	2.798 (0.814 ~ 9.623)
Smoking status (Never smokers)					
Former smokers	1.119 (0.434 ~ 2.882)	1.181 (0.254 ~ 5.319)	1.082 (0.684 ~ 1.713)	0.989 (0.606 ~ 1.612)	1.106 (0.526 ~ 2.322)
Current smokers	1.179 (0.545 ~ 2.551)	1.309 (0.213 ~ 7.937)	1.258 (0.739 ~ 2.140)	1.061 (0.604 ~ 1.864)	1.337 (0.586 ~ 3.050)
Alcohol consumption (Non-regular drinkers)					
Regular drinkers	2.285 (0.929 ~ 5.622)*	2.890 (0.592 ~ 14.098)	1.282 (0.757 ~ 2.173)	1.115 (0.637 ~ 1.949)	1.791 (0.834 ~ 3.846)
Physical activity (≥150 min/w)					
<150 min/w	1.419 (0.781 ~ 2.581)	1.451 (0.304 ~ 6.944)	1.318 (0.870 ~ 1.995)	1.407 (0.913 ~ 2.166)	1.137 (0.595 ~ 2.173)
Rotating night shift work (no)					
Yes	1.658 (0.551 ~ 4.996)	1.289 (0.135 ~ 12.282)	1.067 (0.481 ~ 2.367)	1.150 (0.513 ~ 2.579)	0.655 (0.228 ~ 1.885)

Asterisks denote statistical significance thresholds: * for *p* < 0.10, ** for *p* < 0.05, *** for *p* < 0.01, and **** for *p* < 0.001; the hash symbol (#) indicates that the corresponding variable could not be estimated due to the absence of observed diabetes cases in the firefighter cohort; the Hosmer-Lemeshow goodness-of-fit test yielded *p* > 0.05 for all models; and Nagelkerke *R*^2^ values for the respective models were 0.076/0.150/0.096/0.092/0.052, respectively.

**Figure 2:**
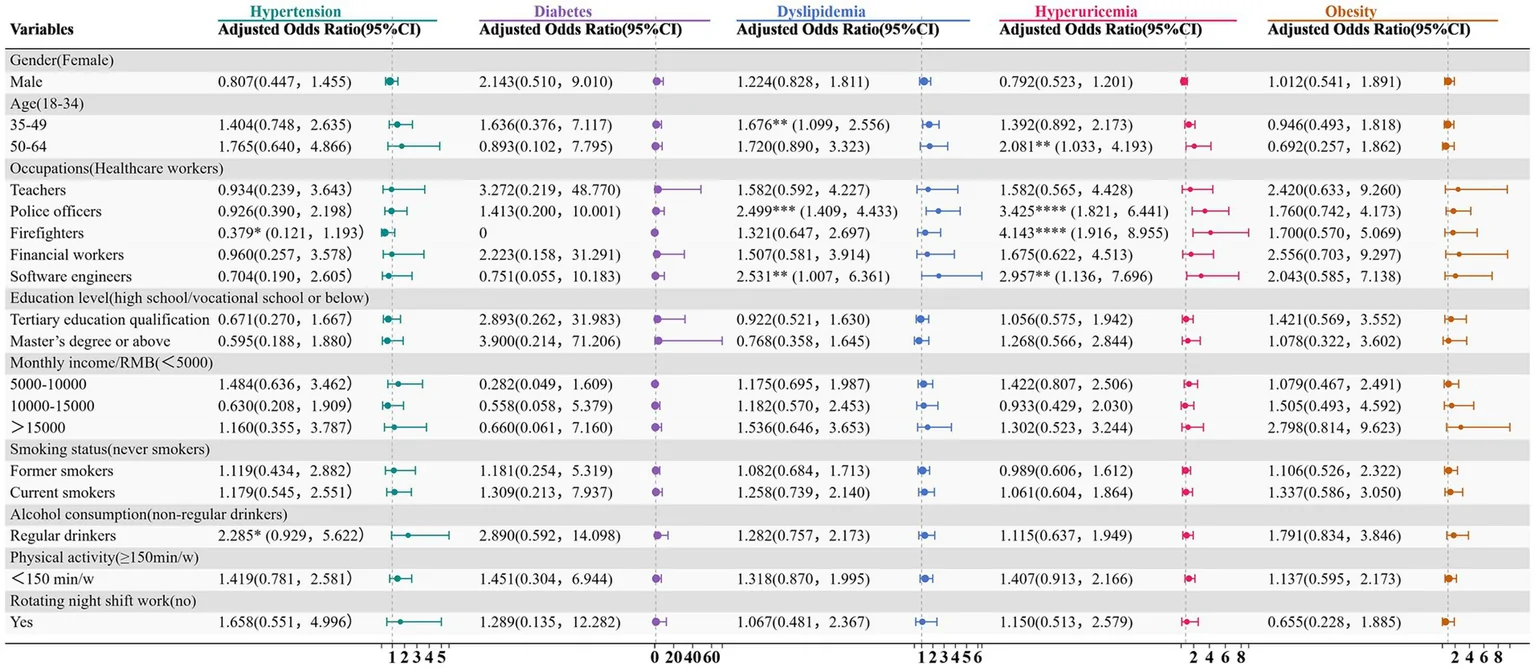
Forest plot of multivariable logistic regression analysis. Filled circles (●) represent the odds ratio (*OR*) for each variable, with the area of the circle reflecting the weight of the variable in the regression model; horizontal lines represent 95% confidence intervals (*CI*). The vertical line at *X* = 1 is the null line, and a line segment fully located to the right of the vertical line indicates the factor is an independent associated factor, while a segment to the left indicates a protective factor. Asterisks denote significance thresholds for *p* values: * for *p* < 0.10, ** for *p* < 0.05, *** for *p* < 0.01, and **** for *p* < 0.001.

### 3.4 Spearman correlation analysis of health indicators

Spearman rank correlation analysis was employed to assess the interrelationships among 16 health indicators in this occupational cohort. As shown in Figure 3, the correlation pattern among blood lipid indicators aligns with the classic features of dyslipidemia associated with metabolic syndrome (MetS). Elevated triglycerides (TG), reduced high-density lipoprotein cholesterol (HDL-C), and elevated low-density lipoprotein cholesterol (LDL-C) are interrelated, and the negative correlation between TG and HDL-C is the most prominent (*r* = −0.54), which may serve as indirect evidence of insulin resistance or MetS risk in this population. Body mass index (BMI) exhibits moderate-strength correlations with multiple metabolic indicators, including decreased TG, reduced HDL-C, elevated uric acid (UA), increased alanine aminotransferase (ALT), and raised systolic/diastolic blood pressure (SBP/DBP), suggesting that obesity acts as the central hub of metabolic dysfunction in this population. Notably, the correlation between BMI and ALT (*r* = 0.51) warrants particular attention, as it may reflect the high prevalence of metabolic dysfunction-associated steatotic liver disease (MASLD) in this population. Blood pressure (especially diastolic blood pressure) shows positive correlations with obesity, TG, and UA; however, most correlation coefficients fall within the range of 0.3–0.4, indicating that these metabolic factors have limited independent contributions to blood pressure variability, although their joint effects may be more substantial. ALT also shows moderate positive correlations with BMI, TG, and UA; combined with the strong correlation between ALT and aspartate aminotransferase (AST) (*r* = 0.76), this strongly indicates a high prevalence of MASLD in this population. ALT can serve as a sensitive indicator for screening liver injury. The strong correlation between uric acid (UA) and creatinine (Cr) (*r* = 0.64) suggests that hyperuricemia is closely associated with reduced renal function in this population, with potential bidirectional causality between the two conditions. Additionally, the moderate correlations between UA and BMI, TG, and ALT further reinforce the perspective that hyperuricemia is a component of metabolic syndrome.

**Figure 3:**
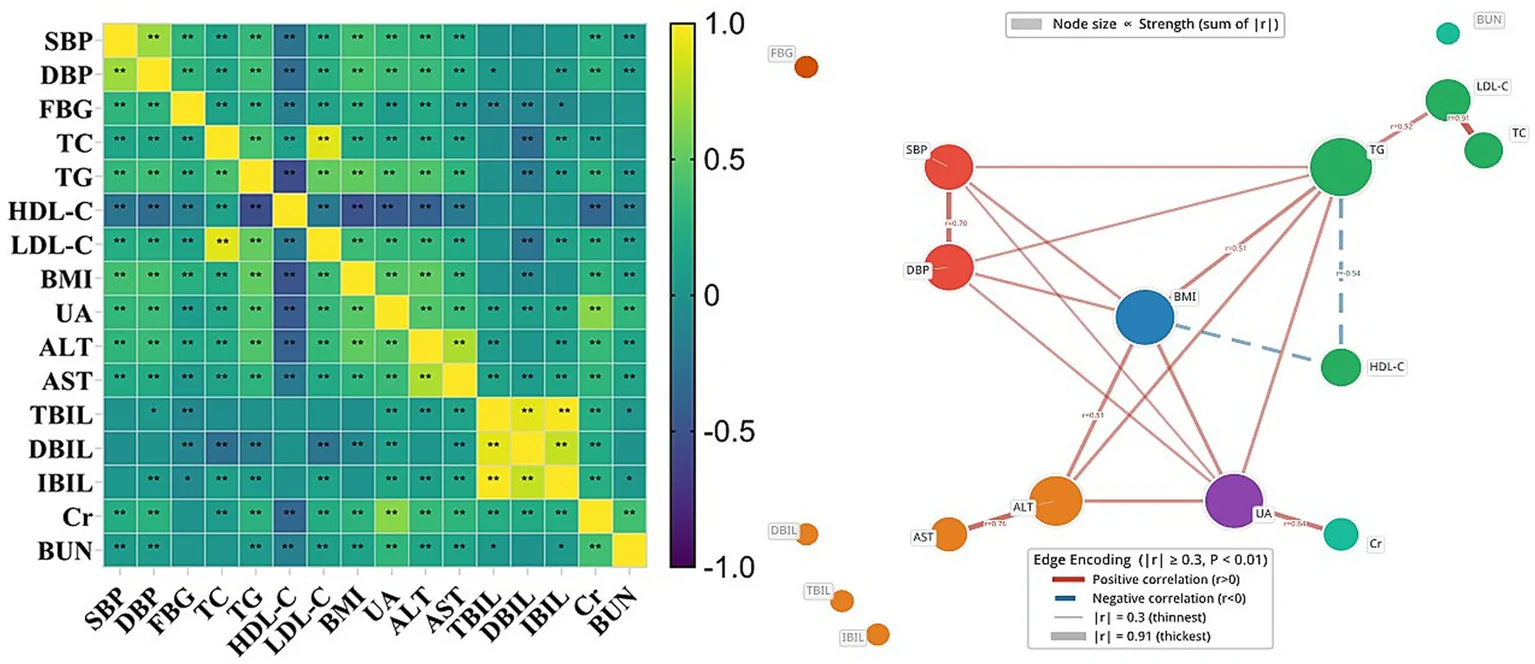
Left panel: Spearman correlation heatmap of 16 health indicators across six occupational groups. Heatmap color intensity represents the Spearman correlation coefficient *r*, with blue indicating negative correlations and yellow indicating positive correlations; darker shade saturation corresponds to the absolute magnitude of |*r*|. Asterisk (*) denotes *p* < 0.05; double asterisk (**) denotes *p* < 0.01. SBP/DBP, systolic/diastolic blood pressure; TG, triglycerides; TC, total cholesterol; LDL-C, low-density lipoprotein cholesterol; HDL-C, high-density lipoprotein cholesterol; UA, uric acid; Cr, creatinine; ALT, alanine aminotransferase; AST, aspartate aminotransferase; BMI, body mass index. Right panel: The figure was generated using R packages. Edges were included when |*r*| ≥ 0.3 (moderate correlation threshold). Network visualization was performed using the igraph package (v1.5.1) with the Fruchterman-Reingold layout algorithm. Correlation network plot of 16 health indicators across six occupational groups (*n* = 1,473). Nodes represent individual health indicators and are colored by predefined category: red = blood pressure, green = blood lipids, purple = uric acid, orange = liver enzymes, blue = body fat, cyan = renal function, brown = glycemic markers. Edges represent statistically significant associations meeting two criteria: Spearman |*r*| ≥ 0.3 and *p* < 0.01. Solid red edges indicate positive correlations, and dashed blue edges indicate negative correlations; edge width is proportional to the magnitude of |*r*|. Node size is proportional to node strength (Strength, defined as the sum of |*r*| values for all edges connected to the node).

## 4 Discussion

In this study, the direct standardization method was employed, using the age structure of the total study sample as the standard population, to calculate the age-standardized prevalence rates (ASRs) of 5 chronic diseases across 6 occupational groups. Crude prevalence rates reflect the actual observed disease burden of each occupation, whereas standardized rates eliminate the influence of age structure differences, enabling a more equitable comparison of inherent disease burdens across different occupational groups (13, 14). Analytical results showed that hyperlipidemia and hyperuricemia were the two chronic diseases with the most pronounced occupational disparities. After age standardization, the ASRs of hyperlipidemia in police officers (ASR = 0.435) and software engineers (ASR = 0.418), as well as the ASRs of hyperuricemia in firefighters (ASR = 0.381) and police officers (ASR = 0.332), were all significantly higher than those in healthcare workers, with corresponding ASR values of 0.218 and 0.143 for the two diseases, respectively. The 95% confidence intervals (CIs) for all pairwise comparisons did not overlap with each other. This finding is highly consistent with the results of the multivariable logistic regression model, which identified police officers, firefighters, and software engineers as being in occupations that serve as independent associated factors for both diseases, collectively indicating that these occupations may be associated with specific metabolic health alterations.

In contrast, occupational disparities in hypertension were relatively small. Although financial practitioners (ASR = 0.179) and teachers (ASR = 0.166) had slightly higher ASR values, the 95% CIs across all occupations extensively overlapped, indicating that the observed differences were statistically non-significant. Similarly, no significant differences in the ASRs of diabetes and obesity were observed across all occupations, and their 95% CIs mutually overlapped. These results suggest that, in the study population, the distribution of disease prevalence for hypertension, diabetes, and obesity may be primarily driven by non-occupation-specific factors including age, genetic background, and individual lifestyle, rather than occupational environment or job nature.

Age standardization effectively revealed the true pattern of disease that was masked by crude surface data. For the teacher group, the crude prevalence rate was overestimated due to a relatively older mean age, and decreased substantially after standardization. In contrast, for the firefighter group, the crude rate was underestimated due to a relatively younger mean age, and the substantially higher actual disease burden related to hyperlipidemia and hyperuricemia was revealed after standardization. This contrast clearly demonstrates that implementing age standardization is an essential step when comparing health status across cross-occupational populations, to avoid drawing misleading conclusions. Of 2,150 questionnaire invitations distributed, 1,524 valid responses were obtained (response rate 70.9%). Non-respondents were slightly older (mean age 41.2 vs. 38.7 years, *p* = 0.012) and more likely to be male (68% vs. 63%, *p* = 0.034), but did not differ significantly in occupational distribution (*p* = 0.21). Because non-respondents were older and more frequently male, both correlates of higher chronic disease prevalence, the true disease burden in the underlying occupational population may be underestimated in our sample. Furthermore, the healthy worker effect and self-selection into health examinations at a tertiary hospital may have compounded this downward bias. We therefore interpret our prevalence estimates as conservative and recommend cautious generalization to the broader occupational population.

After multivariable adjustment, police officers, firefighters, and software workers had significantly higher odds of hyperlipidemia and hyperuricemia compared with healthcare workers (the reference group). Among them, firefighters had the most prominent correlates of hyperuricemia (OR = 4.14), while police officers showed significantly elevated correlates of both hyperlipidemia (OR = 2.50) and hyperuricemia (OR = 3.43). These associations remained independent of traditional factors including age, sex, income, smoking, and alcohol consumption. The 35–49 years age group was an independent correlate of hyperlipidemia, while the 50–64 years age group was an independent correlate of hyperuricemia. However, no significant association between age and hypertension, diabetes, or obesity was observed in this study (which may be attributable to sample size or age distribution). Traditional behavioral factors (smoking, alcohol consumption, physical activity, and rotating night shifts) did not achieve statistical significance in this study. Although active alcohol consumption showed a trend toward association with hypertension (*p* < 0.10) and insufficient physical activity showed a trend across all included diseases (OR > 1 for all conditions but *p* > 0.05), the sample size of this study may have been insufficient to detect these factors with moderate effect sizes, and the true effects of these factors may be more pronounced in other subgroups. Consistent with recent evidence that unfavorable social determinants of health, particularly low education, unemployment or retirement, and limited income, are independently associated with cardiometabolic syndrome progression in the Chinese population, the occupational disparities in chronic disease prevalence and metabolic comorbidity networks observed in our study likely reflect structured social stratification across occupations (15). Future studies should consider incorporating unmeasured factors such as dietary pattern, physical activity intensity (rather than exercise duration alone), psychological stress, sleep quality, and genetic susceptibility (16–19). Unmeasured diet, psychological stress, sleep quality, and genetic susceptibility may confound the observed associations. Pro-inflammatory diets are linked to dyslipidemia (20); sleep disturbances are associated with metabolic traits (21); and polygenic risk scores explain substantial variation in obesity (22). These unmeasured factors likely act as confounders or effect modifiers of the observed associations. Future studies should integrate these measures to isolate occupational effects.

Correlation analysis of health indicators in this occupational population revealed a tightly interwoven metabolic covariation network. Among them, TG, HDL-C, BMI, UA and ALT formed moderate positive/negative correlation clusters (|r| between 0.44 and 0.54), which strongly suggested that there was a significant aggregation of metabolic abnormalities in this population. In this network, the negative correlation between TG and HDL-C (*r* = −0.54) and the positive correlation between BMI and ALT (*r* = 0.51) represented the most characteristic association patterns. Notably, as the central node of this network, BMI exhibited moderate positive correlations with TG, UA, ALT, and SBP/DBP, and a moderate negative correlation with HDL-C, highlighting its core role in integrating multiple metabolic abnormalities and indicating that weight management should serve as a key entry point for health interventions targeting this population. Liver enzyme levels are highly correlated with metabolic disorders, with correlation coefficients between ALT and BMI, TG, and UA all falling between 0.44 and 0.51. Moreover, the extremely strong correlation between ALT and AST (*r* = 0.76) collectively suggests that MASLD may have a high potential prevalence in this population. Uric acid levels also demonstrate strong associations with renal function indicators; specifically, the correlation coefficient between UA and creatinine (Cr) reaches as high as 0.64, making it one of the strongest cross-system associations observed in this study. This finding is consistent with existing evidence that hyperuricemia is a correlate of renal diseases, suggesting that uric acid level monitoring and renal function assessment should be strengthened for this population. In contrast, the associations between blood pressure (SBP/DBP) and core metabolic indicators (e.g., BMI, TG, UA) are relatively weak (*r* ≈ 0.3–0.4), with their intensity lower than that of intra-metabolic cluster associations, implying that blood pressure variation may be more strongly influenced by non-metabolic factors such as age, dietary sodium intake, and autonomic nervous system regulation (23–26). The internal correlation pattern of blood lipids conforms to conventional understanding; the extremely strong correlation between TC and LDL-C (*r* = 0.91) validates the reliability of this dataset, whereas the negative correlation between TG and HDL-C (*r* = −0.54) further corroborates the universality of this relationship as a core feature of metabolic syndrome. Regarding the zero diabetes cases observed among firefighters, this phenomenon is attributable to the combined effect of the relatively small sample size within this subgroup (*n* = 200) and the pronounced healthy worker effect inherent to this occupation. Firefighters undergo rigorous annual physical fitness and medical screenings, which systematically exclude individuals with established metabolic disorders. Importantly, sensitivity analysis excluding firefighters from the diabetes model confirmed that the odds ratios for all other occupational groups remained non-significant and materially unchanged (Supplementary Table S4). Therefore, the zero-event does not alter the overall conclusion that no statistically significant occupational disparity in diabetes was detected. Future studies with larger sample sizes or longitudinal designs are warranted to further explore occupational diabetes risk in physically demanding professions.

Because body mass index (BMI) is a well-established correlate of metabolic diseases and was identified as a central hub in our correlation network, we repeated all five logistic regression models with BMI included as an additional continuous covariate. BMI was a significant independent correlate in every model (all *p* < 0.01). As shown in Supplementary Table S6, occupational odds ratios changed by less than 10% in either direction. Consistent with expectation, occupations with higher mean BMI (police officers, software engineers) showed modest decreases in OR (−4.3 to −6.2%), while firefighters, with the lowest mean BMI due to intense physical demands, showed slight increases (+1.1% to +1.6%); teachers and financial workers (BMI ≈ healthcare workers) showed negligible changes (<1%). All previously significant associations remained statistically significant, confirming that the observed occupational disparities are robust to adjustment for BMI.

This study has certain limitations. The model’s Nagelkerke R^2^ is low, indicating that the measured variables only account for a portion of the variation in disease prevalence. The model’s Nagelkerke *R*^2^ is bounded by the outcome event rate. However, the value observed in the present study falls within the range expected for comparable studies, and the *p*-value for overall model fit is greater than 0.05, indicating good model calibration. Furthermore, the cross-sectional design precludes establishment of causality, and self-reported data may be subject to recall bias. Although occupation may exert effects on metabolic health through behavioral and environmental pathways, pre-existing metabolic disorders may also shape occupational selection, meaning that reverse causality cannot be ruled out. The study sample was drawn from a single center, so the participants may differ from those who do not undergo regular health examinations or who are examined elsewhere in terms of health awareness, accessibility of medical services, and socioeconomic status, raising the possibility of a “healthy-worker” effect (27, 28). Consequently, the results chiefly reflect the characteristics of the specific occupational population served by this health-examination center, and extrapolation to broader occupational groups should be undertaken with caution. Extrapolation of the results to non-physical examination population or non-employed population should be cautious. Future studies should extend beyond cross-sectional snapshots to adopt trajectory-aware multi-organ frameworks, such as the Health Galaxy model (29), to characterize the longitudinal evolution of occupational metabolic disparities across organ systems. Future multicenter, community-based, or industry-wide registry studies are needed to confirm these findings.

## 5 Conclusion

This study employed a cross-sectional survey of working-age individuals from six typical occupations (healthcare workers, teachers, police officers, firefighters, software engineers, and financial workers), integrating age-standardized prevalence, multivariable logistic regression, and correlation analysis of examination indices to systematically assess the association between occupational differences and chronic-disease prevalence, as well as its potential metabolic basis.

The principal finding was that occupational differences in hyperlipidemia and hyperuricemia were most pronounced; after age standardization, police officers, software engineers, and firefighters exhibited significantly higher prevalence than healthcare workers, and multivariable analysis confirmed these occupations as independent associated factors (OR = 2.50–4.14). Age standardization is a crucial methodological safeguard for cross-occupational health comparisons, effectively correcting for age-structure confounding and preventing bias introduced by crude rates. The core examination indices in this occupational cohort form a tightly coupled metabolic covariance network, with BMI occupying a hub position and being broadly associated with multiple metabolic abnormalities; the strong correlation between UA and Cr, and the extremely strong correlation between ALT and AST, respectively indicate a potential for renal injury associated with hyperuricemia and a high latent burden of MASLD.

This study provides epidemiological evidence from a multi-occupational Lanzhou population for understanding the link between occupation and chronic-disease prevalence, identifies metabolic-health correlates specific to certain occupations, and elucidates the central integrative role of obesity within the multimorbidity network. These findings offer valuable reference for developing occupation-specific, precision-oriented strategies for chronic-disease prevention and health promotion in the region.

## Data Availability

Data are available from the corresponding author upon reasonable request. The data are not publicly available due to privacy or ethical restriction.

## Supplementary material

Supplementary Material S1 Self-developed structured questionnaire on the health status of the occupational population.

## Glossary

ASRs: Age-standardized prevalence rates
SD: Standard deviation
OR: Odds Ratio
BMI: Body Mass Index
SPSS: Statistical Package for the Social Sciences
χ^2^: Chi-square
CI: Confidence Interval
MetS: Metabolic syndrome
MASLD: Metabolic dysfunction-associated steatotic liver disease
DALYs: Disability-adjusted life years
LMICs: Low-and middle-income countries
SBP: Systolic Blood Pressure
DBP: Diastolic Blood Pressure
FBG: Fasting Blood Glucose
TC: Total Cholesterol
TG: Triglycerides
HDL-C: High-Density Lipoprotein Cholesterol
LDL-C: Low-Density Lipoprotein Cholesterol
BMI: Body Mass Index
UA: Uric Acid
ALT: Alanine Transaminase
AST: Aspartate Transaminase
TBIL: Total Bilirubin
DBIL: Direct Bilirubin
IBIL: Indirect Bilirubin
Cr: Creatinine
BUN: Blood Urea Nitrogen
