# Supplementary material for: Occupational disparities in chronic disease prevalence and metabolic covariation: a cross-sectional study of six occupational groups in Lanzhou
Source: Front Public Health. 2026 Sep 21;14:1950073. doi: 10.3389/fpubh.2026.1950073 (PMC13635345; doi:10.3389/fpubh.2026.1950073)
Supplement: Supplementary file 1 [file Supplementary_file_1.docx]

**Supplementary Table S1:** Sensitivity analysis excluding teachers (n=156) and financial workers (n=193)

| Model | Original OR (95% CI) | Sensitivity OR* (95% CI) | P |
| --- | --- | --- | --- |
| Dyslipidemia |  |  |  |
| Police officers | 2.499 (1.409–4.433) | 2.483 (1.391–5.218) | 0.044 |
| Software engineers | 2.531 (1.007–6.361) | 2.526 (1.002–7.059) | 0.048 |
| Hyperuricemia |  |  |  |
| Police officers | 3.425 (1.821–6.441) | 3.401 (1.807–7.424) | 0.009 |
| Firefighters | 4.143 (1.916–8.955) | 4.128 (1.903–9.632) | 0.008 |
| Software engineers | 2.957 (1.136–7.696) | 2.944 (1.132–8.685) | 0.036 |

**Supplementary Table S2:** Variance Inflation Factors (VIF) for independent variables in the logistic regression models.

| Variable | Tolerance | VIF |
| --- | --- | --- |
| Gender | 0.437 | 2.291 |
| Age | 0.373 | 2.680 |
| Occupation | 0.352 | 2.843 |
| Monthly income/RMB | 0.390 | 2.562 |
| Smoking status | 0.239 | 4.192 |
| Alcohol consumption | 0.320 | 3.121 |
| Physical activity | 0.460 | 2.173 |
| Rotating night shift | 0.360 | 2.775 |

Note: Collinearity statistics were derived from the final multivariable logistic regression model predicting the presence of metabolic syndrome and its components. All Variance Inflation Factor (VIF) values are below the conventional threshold of 5, and Tolerance values are above 0.1, indicating no severe multicollinearity among the predictor variables.

**Supplementary Table S3:** Age-standardized prevalence (ASR) using the Gansu Province 2020 Population Census (7th National Census) external standard population, compared with the internal standard population.

| Disease | Occupation | CR(%) | ASR(int)(%) | 95%CI(int) | ASR(ext)(%) | 95%CI(ext) | Δ% |
| --- | --- | --- | --- | --- | --- | --- | --- |
| Hypertension | Healthcare workers | 9.8 | 8.8 | (7.5-13.8) | 15.4 | (9.3-21.5) | 75.00% |
| Hypertension | Teachers | 18.5 | 16.6 | (10.7-22.4) | 17.7 | (11.9-23.6) | 6.60% |
| Hypertension | Police officers | 12 | 10.8 | (6.5-15.1) | 13.2 | (8.4-18.1) | 22.20% |
| Hypertension | Firefighters | 5.1 | 4.6 | (1.7-7.5) | 11.8 | (-15.3-38.9) | 156.50% |
| Hypertension | Software engineers | 10.5 | 9.4 | (6.2-12.6) | 21.0 | (4.8-37.3) | 123.40% |
| Hypertension | Financial workers | 20 | 17.9 | (12.5-23.3) | 22.2 | (16.3-28.0) | 24.00% |
| Diabetes | Healthcare workers | 2.5 | 2.2 | (0.8-3.7) | 4.3 | (0.6-8.1) | 95.50% |
| Diabetes | Teachers | 7 | 6.3 | (2.4-10.1) | 6.7 | (2.8-10.7) | 6.30% |
| Diabetes | Police officers | 3 | 2.7 | (0.4-5.0) | 3.4 | (0.7-6.0) | 25.90% |
| Diabetes | Firefighters | N/A | N/A | N/A | N/A | N/A | -- |
| Diabetes | Software engineers | 2.5 | 2.2 | (0.6-3.9) | 5.9 | (-4.7-16.5) | 168.20% |
| Diabetes | Financial workers | 4.5 | 4 | (1.2-6.8) | 5.1 | (1.8-8.3) | 27.50% |
| Hyperlipidemia | Healthcare workers | 24.3 | 21.8 | (17.8-25.8) | 31.2 | (24.1-38.3) | 43.10% |
| Hyperlipidemia | Teachers | 41.5 | 37.2 | (29.8-44.7) | 40.4 | (32.7-48.1) | 8.60% |
| Hyperlipidemia | Police officers | 48.5 | 43.5 | (36.8-50.2) | 51.4 | (44.9-57.8) | 18.20% |
| Hyperlipidemia | Firefighters | 35 | 31.4 | (25.0-37.7) | 53.4 | (25.8-81.1) | 70.10% |
| Hyperlipidemia | Software engineers | 46.6 | 41.8 | (36.6-47.0) | 67.0 | (61.5-72.5) | 60.30% |
| Hyperlipidemia | Financial workers | 37.5 | 33.6 | (27.1-40.2) | 39.9 | (33.1-46.6) | 18.70% |
| Hyperuricemia | Healthcare workers | 16 | 14.3 | (10.9-17.8) | 18.6 | (12.5-24.7) | 30.10% |
| Hyperuricemia | Teachers | 29.5 | 26.5 | (19.6-33.3) | 28.7 | (21.3-36.1) | 8.30% |
| Hyperuricemia | Police officers | 37 | 33.2 | (26.7-39.6) | 38.3 | (31.6-45.1) | 15.40% |
| Hyperuricemia | Firefighters | 42.5 | 38.1 | (31.5-44.7) | 55.3 | (24.9-85.8) | 45.10% |
| Hyperuricemia | Software engineers | 34.9 | 31.3 | (26.3-36.3) | 43.6 | (26.8-60.5) | 39.30% |
| Hyperuricemia | Financial workers | 30.5 | 27.4 | (21.1-33.6) | 31.7 | (25.1-38.4) | 15.70% |
| Obesity | Healthcare workers | 9.3 | 8.3 | (5.6-11.1) | 9.3 | (5.0-13.5) | 12.00% |
| Obesity | Teachers | 13 | 11.7 | (6.6-16.7) | 12.8 | (7.2-18.4) | 9.40% |
| Obesity | Police officers | 13.6 | 12.2 | (7.6-16.7) | 13.6 | (8.7-18.4) | 11.50% |
| Obesity | Firefighters | 10.5 | 9.4 | (5.4-13.5) | 10.7 | (-9.8-31.1) | 13.80% |
| Obesity | Software engineers | 12.3 | 11 | (7.6-14.4) | 12.4 | (1.3-23.4) | 12.70% |
| Obesity | Financial workers | 13.5 | 12.1 | (7.5-16.7) | 13.5 | (8.6-18.4) | 11.60% |

Abbreviations: CR = crude prevalence; ASR(int) = age-standardized prevalence using the internal standard (age distribution of the full study sample: 18–34 years 54.9%, 35–49 years 32.8%, 50–64 years 12.3%); ASR(ext) = age-standardized prevalence using the Gansu Province 2020 Census external standard (18–34 years 34.13%, 35–49 years 32.20%, 50–64 years 33.68%). Weights for the external standard were derived from the Gansu Province 7th National Census Bulletin.

**Supplementary Table S4:** Sensitivity analysis of diabetes logistic regression model excluding firefighters (n=200).

| Occupation | Original OR (95% CI) (n=1,473) | Sensitivity OR (95% CI) (n=1,273) | ΔOR(%) | Significance |
| --- | --- | --- | --- | --- |
| Healthcare workers | – | – | – | – |
| Teachers | 3.272 (0.219–48.770) | 2.978 (0.197–44.967) | −9.0% | NS |
| Police officers | 1.413 (0.200–10.001) | 1.856 (0.259–13.312) | 31.4% | NS |
| Financial workers | 2.223 (0.158–31.291) | 2.056 (0.145–29.189) | −7.5% | NS |
| Software engineers | 0.751 (0.055–10.183) | 0.899 (0.065–12.374) | 19.7% | NS |

Note: Firefighters excluded due to zero observed diabetes cases. All models adjusted for gender, age, education, income, smoking, alcohol, physical activity, and rotating night shift. NS = not significant (P>0.05). %Δ OR = (sensitivity OR − original OR) / original OR × 100%.

**Supplementary Table S5:** Correlation network threshold

| Threshold(\|r\|) | Number of edges |  |
| --- | --- | --- |
| ≥0.3 | 19 | All 5 key edges (BMI-TG, BMI-ALT, BMI-UA, TG-HDL-C, UA-Cr, ALT-AST) retained |
| ≥0.4 | 14 | BMI-TG, BMI-ALT, BMI-UA, TG-HDL-C, UA-Cr, ALT-AST retained; Lost weaker edges: BMI-SBP, BMI-DBP, etc. |
| ≥0.5 | 8 | BMI-TG, BMI-ALT, TG-HDL-C, UA-Cr, ALT-AST retained; Lost BMI-UA and several moderate edges |

The core network structure, BMI as a central hub connecting TG, ALT, UA, and HDL-C, plus the strong UA-Cr and ALT-AST pairs, remained stable across all three thresholds. Only peripheral weak edges (|r|<0.4) were lost, confirming that the choice of |r|≥0.3 is reasonable and does not drive the main conclusions.

**Supplementary Table S6:** Sensitivity analysis adjusting for BMI in logistic regression models

| Disease | Occupation | Main OR (95% CI) | BMI-adj OR (95% CI) | Δ OR(%) |
| --- | --- | --- | --- | --- |
| Dyslipidemia | Healthcare(ref) | 1.000 | 1.000 | – |
|  | Teachers | 1.582 (0.592–4.227) | 1.571 (0.583–4.232) | −0.7% |
|  | **Police officers** | **2.499 (1.409–4.433)** | **2.345 (1.311–4.195)** | **−6.2%** |
|  | Firefighters | 1.321 (0.647–2.697) | 1.338 (0.651–2.749) | +1.3% |
|  | Financial workers | 1.507 (0.581–3.914) | 1.498 (0.574–3.909) | −0.6% |
|  | **Software engineers** | **2.531 (1.007–6.361)** | **2.405 (0.950–6.092)** | **−5.0%** |
| Hyperuricemia | Healthcare(ref) | 1.000 | 1.000 | – |
|  | Teachers | 1.582 (0.565–4.428) | 1.574 (0.558–4.437) | −0.5% |
|  | **Police officers** | **3.425 (1.821–6.441)** | **3.225 (1.702–6.115)** | **−5.8%** |
|  | **Firefighters** | **4.143 (1.916–8.955)** | **4.202 (1.936–9.126)** | **+1.4%** |
|  | Financial workers | 1.675 (0.622–4.513) | 1.664 (0.615–4.505) | −0.7% |
|  | **Software engineers** | **2.957 (1.136–7.696)** | **2.818 (1.076–7.382)** | **−4.7%** |
| Hypertension | Healthcare(ref) |  |  |  |
|  | Teachers | 0.934 (0.239–3.643) | 0.928 (0.235–3.665) | −0.6% |
|  | Police officers | 0.926 (0.390–2.198) | 0.872 (0.362–2.101) | −5.8% |
|  | **Firefighters** | **0.379 (0.121–1.193)** | **0.385 (0.122–1.217)** | **+1.6%** |
|  | Financial workers | 0.960 (0.257~3.578) | 0.957 (0.249–3.937) | −0.3% |
|  | Software engineers | 0.704 (0.190–2.605) | 0.670 (0.178–2.524) | −4.8% |
| Diabetes | Healthcare(ref) |  |  |  |
|  | Teachers | 3.272 (0.219–48.770) | 3.265 (0.217–49.185) | −0.2% |
|  | Police officers | 1.413 (0.200–10.001) | 1.342 (0.188–9.578) | −5.0% |
|  | Firefighters | – | – | – |
|  | Financial workers | 2.223 (0.158~31.291) | 2.216 (0.151–27.281) | −0.3% |
|  | Software engineers | 0.751 (0.055–10.183) | 0.719 (0.052–9.940) | −4.3% |
| Obesity | Healthcare(ref) |  |  |  |
|  | Teachers | 2.420 (0.633–9.260) | 2.412 (0.626–9.295) | −0.3% |
|  | Police officers | 1.760 (0.742–4.173) | 1.662 (0.691–3.996) | −5.6% |
|  | Firefighters | 1.700 (0.570–5.069) | 1.718 (0.573–5.152) | +1.1% |
|  | Financial workers | 1.507 (0.581–3.914) | 1.496 (0.574–4.418) | −0.7% |
|  | Software engineers | 2.556 (0.703~9.297) | 1.950 (0.551–6.902) | −4.6% |

%Δ OR = (BMI-adjusted OR − main OR) / main OR × 100%. Negative = OR decreased after BMI adjustment; positive = OR increased. All changes <10%, and all significant associations remained significant (P<0.05). BMI was a significant independent correlate in all five models (all P<0.01).

**Supplementary Table S7:** Descriptive statistics of 16 health indicators across six occupational groups.

| Indicator | Healthcare workers (n=400) | Teachers (n=156) | Firefighters (n=200) | Financial workers (n=193) | Software engineers (n=324) | Police officers (n=200) | P |
| --- | --- | --- | --- | --- | --- | --- | --- |
| BMI (kg/m²) | 23.5 ± 3.1 | 24.8 ± 3.5 | 23.1 ± 2.8 | 24.6 ± 3.3 | 24.0 ± 3.2 | 25.2 ± 3.0 | <0.001 |
| SBP (mmHg) | 118.5 ± 15.2 | 124.3 ± 16.8 | 119.0 ± 13.9 | 123.7± 16.1 | 120.2 ± 14.8 | 122.1 ± 14.5 | <0.001 |
| DBP (mmHg) | 76.2 ± 10.1 | 79.5 ± 11.3 | 75.9 ± 9.4 | 79.1 ± 10.8 | 77.0 ± 10.2 | 78.8 ± 9.7 | <0.001 |
| TC (mmol/L) | 4.5 ± 0.9 | 4.8 ± 0.9 | 4.5 ± 0.8 | 4.7 ± 0.9 | 4.6 ± 0.8 | 4.8 ± 0.8 | <0.001 |
| TG (mmol/L) | 1.3 ± 0.7 | 1.5 ± 0.8 | 1.4 ± 0.7 | 1.6 ± 0.8 | 1.8 ± 1.1 | 1.8 ± 0.9 | <0.001 |
| HDL-C (mmol/L) | 1.4 ± 0.3 | 1.3 ± 0.3 | 1.3 ± 0.3 | 1.3 ± 0.3 | 1.3 ± 0.3 | 1.2 ± 0.3 | <0.001 |
| LDL-C (mmol/L) | 2.6 ± 0.7 | 2.8 ± 0.8 | 2.6 ± 0.7 | 2.8 ± 0.7 | 2.7 ± 0.7 | 2.9 ± 0.7 | <0.001 |
| UA (µmol/L) | 335.2 ± 82.5 | 378.6 ± 90.3 | 386.2 ± 85.4 | 380.1 ± 89.2 | 384.0 ± 78.6 | 408.5 ± 88.7 | <0.001 |
| Cr (µmol/L) | 72.3 ± 15.6 | 75.8 ± 16.2 | 76.1 ± 15.3 | 74.9 ± 15.8 | 73.6 ± 15.1 | 78.5 ± 14.9 | <0.001 |
| BUN (mmol/L) | 5.1 ± 1.3 | 5.3 ± 1.4 | 5.1 ± 1.3 | 5.2 ± 1.4 | 5.2 ± 1.3 | 5.45 ± 1.4 | 0.035 |
| ALT (U/L) | 22.5 ± 12.3 | 26.8 ± 15.1 | 24.1 ± 13.2 | 27.5 ± 14.6 | 25.0 ± 13.5 | 28.2 ± 14.8 | <0.001 |
| AST (U/L) | 21.8 ± 9.5 | 24.2 ± 11.2 | 22.5 ± 10.0 | 24.8 ± 11.0 | 23.2 ± 10.3 | 25.0 ± 10.8 | <0.001 |
| TBIL (µmol/L) | 12.5 ± 4.8 | 13.2 ± 5.1 | 12.8 ± 4.9 | 13.0 ± 5.2 | 12.6 ± 4.7 | 13.8 ± 5.0 | 0.062 |
| DBIL (µmol/L) | 3.8 ± 1.5 | 4.0 ± 1.6 | 3.9 ± 1.4 | 4.0 ± 1.6 | 3.9 ± 1.5 | 4.2 ± 1.5 | 0.071 |
| IBIL (µmol/L) | 8.7 ± 3.5 | 9.2 ± 3.8 | 8.9 ± 3.6 | 9.0 ± 3.9 | 8.7 ± 3.4 | 9.6 ± 3.7 | 0.045 |
| FPG (mmol/L) | 5.1 ± 0.8 | 5.3 ± 0.9 | 5.1 ± 0.8 | 5.3 ± 0.9 | 5.2 ± 0.8 | 5.2 ± 0.8 | <0.001 |

Data are presented as mean ± standard deviation. P-values were obtained from one-way ANOVA or Kruskal-Wallis test (for non-normally distributed variables).
